# Polysaccharides, Total Phenolic, and Flavonoid Content from Different Kenaf (*Hibiscus cannabinus* L.) Genotypes and Their Antioxidants and Antibacterial Properties

**DOI:** 10.3390/plants10091900

**Published:** 2021-09-14

**Authors:** Ziggiju Mesenbet Birhanie, Aiping Xiao, Dawei Yang, Siqi Huang, Chao Zhang, Lining Zhao, Liangliang Liu, Jianjun Li, Anguo Chen, Huijuan Tang, Li Chang, Gen Pan, Cuiping Zhang, Ashok Biswas, Susmita Dey, Defang Li, Yong Deng

**Affiliations:** Institute of Bast Fiber Crops, Chinese Academy of Agricultural Sciences, Changsha 410000, China; zegje23@gmail.com (Z.M.B.); xiaoaiping@caas.cn (A.X.); 18317826602@163.com (D.Y.); huangsiqi@caas.cn (S.H.); zhangchao_20180604@163.com (C.Z.); csbtzln@163.com (L.Z.); liuliangliang@caas.cn (L.L.); lijianjun006@163.com (J.L.); CAGIBFC@126.com (A.C.); tanghuijuan@caas.cn (H.T.); changli519@163.com (L.C.); pangen@caas.cn (G.P.); hncszcp@126.com (C.Z.); ashok.ag1sau@gmail.com (A.B.); susmita.ag4sau@gmail.com (S.D.)

**Keywords:** *Hibiscus cannabinus*, antioxidant activity, antibacterial activity, polysaccharide, phenolic content, flavonoid content

## Abstract

Kenaf (*Hibiscus cannabinus* L.) is a valuable plant with a potential health benefit because of its extensive bioactive compounds. Leaf extracts of 33 kenaf genotypes were investigated for their polysaccharide, total phenolic, and flavonoid content. The antioxidant properties were evaluated by 2,2-Diphenyl-1-picrylhydrazyl (DPPH), 2,2′-azinobis (3-ethylbenzothiazoline-6-sulfonic acid (ABTS), and ferric reducing antioxidant potential (FRAP) assays. Antimicrobial capacity was also assessed against *Staphylococcus aureus* and *Escherichia coli* using a disc diffusion assay. The polysaccharide content varied from 6.45–16.12 mg glucose per g DW. Total phenolic and flavonoid content ranged from 6.03–21.15 mg GAE/g DW and 1.55–9.24 mg RE/g DW, respectively. Similarly, varied values in the range 20.55–79.99% of inhibition by DPPH, 56.28–88.30% of inhibition by ABTS and 1.26–5.08 mmol Fe^2+^/g DW by FRAP assays were obtained for antioxidants of the genotype extracts. Extracts from CS4 and CS2 genotypes had the highest antioxidant activities. Kenaf leaves exhibited antibacterial activity against *Staphylococcus aureus* and *Escherichia coli*. Strong correlation was found between antioxidant activity with polysaccharide (DPPH, r = 0.893; ABTS, r = 0.819; FRAP, r = 0.864) and total phenolic content (DPPH, r = 0.850; ABTS, r = 0.959; FRAP, r = 0.953). The results suggested that the kenaf leaves could be used as a natural antioxidants and antimicrobial in food industries.

## 1. Introduction

Even with the improvement in living standards, large numbers of people suffer from non-infectious diseases associated with free radicals which could be controlled by antioxidants [1]. Antioxidants play a significant role in preventing degenerative diseases, including cancer, cardiovascular, and neurological triggered by free radicals generated during cellular metabolism [2,3,4].

Plant bioactive constituents have been a major area of interest to scientists in recent years due to their great potential in human health. Phenolic and flavonoid compounds are part of the plant bioactive substances exhibiting antioxidant and antibacterial properties [5,6,7] and are widely distributed in vegetables, fruits, and herbaceous medicinal plants [8]. Similarly, polysaccharides from plants have been proven to possess a good biological activity, such as immunity regulation, antitumor activity [9], antioxidant activity [10], and antibacterial activity [11].

Kenaf (*Hibiscus cannabinus* L.) is a herbaceous, dicotyledonous plant originated from Africa with a wide distribution encouraged by climate adaptation [12]. It has been cultivated for years for textiles, paper, and cordage. Kenaf leaves have many important medicinal properties, including antioxidants, anti-inflammatory, anticancer, aphrodisiacs, analgesic, and hepatoprotective activities [13,14,15]. Kenaf leaf is used in traditional African medicine to treat anemia and Guinea worms disease [16]. Additionally, in ayurvedic medicine, researchers have found kenaf leaf helpful against fatigue, blood, bilious, diabetes, and throat diseases [13,17]. There is also evidence that kenaf extract is one of the keys in healing scurvy and jaundice, exciting the stomach and improving its action [17]. It is also used in the pharmaceuticals and food industry as a value-added ingredient [18,19,20]. The kenaf leaves can be used to make healthy beverages as well as functional additives for bakery products [21]. Additionally, tea prepared from kenaf leaves has been recognized as a supplement to the human diet [18]. These medicinal benefits are revealed due to the presence of polysaccharide, flavonoid [13,22], and phenolic [14,17] compounds with antioxidant and antibacterial activities in the kenaf plant [8,12]. A previous study reported that the polysaccharides extracted from kenaf seed have a cholesterol lowering effect in rats [23].

To the best of our knowledge, no information has been published about polysaccharides from kenaf leaves and a few scientific reports have been found on the variation of total phenolic, flavonoid content, antioxidant, and antibacterial activities of leaves’ extracts from different kenaf genotypes. This research was therefore aimed at determining the polysaccharides, total phenolic, and flavonoid contents in the leaves of 33 kenaf genotypes and evaluate their antioxidant and antibacterial properties.

## 2. Results and Discussion

### 2.1. Polysaccharide Content of Kenaf Leaf Extracts

The polysaccharide content varied from 6.45–16.12 mg glucose/g DW as showed in Table 1. CS4 (16.12 mg glucose/g DW) had the highest polysaccharide contents, followed by CS2 (16.12 mg glucose/g DW) and CS33 (14.57 mg glucose/g DW); however, CS18 had the lowest polysaccharide content among the tested kenaf genotypes.

Polysaccharides are abundant in plants as bioactive substances, but little is known about the polysaccharide content in kenaf. When comparing values in this study with other medicinal plants, kenaf leaves have a comparable amount of polysaccharide. As reported in a previous study, the polysaccharide content in 49 edible macro-fungi ranging from 2.440 to 14.145 mg glucose/g [24] and 8.13–22.56 mg/g dry weight g in *Callerya speciosa* Champ [25]. To the best of our knowledge, this report is the first to determine the polysaccharide content in kenaf leaf. The results showed that kenaf has a high protentional for use in the food and pharmaceutical industries.

### 2.2. Total Phenolic Content of Kenaf Leaf Extracts

Total phenolic content of each kenaf genotype were determined, and the results are summarized in (Table 1). Significant differences in the range of 6.03–21.15 mg GAE/g DW were observed among the genotypes. The highest total phenolic content (21.15 mg GAE/g DW) was found in CS4 extract followed by CS2 extract (19.40 mg GAE/g DW), CS27 extract (16.81 mg GAE/g DW), CS33 extract (16.63 mg GAE/g DW), and CS16 extract (16.34 mg GAE/g DW). The lowest total phenolic content (6.03 mg GAE/g DW) was obtained in the CS18 extract.

As Ryu et al. reported, total phenolic content of three kenaf varieties ranged from 17.4–28 mg GAE/g dry weight [26]. In the other study, the total phenolic content varied from 19 to 26 mg GAE/g dry weight for two varieties [17]. Similarly, Zhen et al. reported that total phenolic content in the leaves of 22 roselle (*H. sabdariffa*) accessions varied from 19.0–30.0 mg GAE /g depending on the cultivar [27]. The total phenol content obtained from this study (6.03–21.15 mg GAE/g DW) is comparable to the results found in extracts of 56 commonly consumed vegetables (4.99–23.27 mg GAE/g) [28]. When the results of this study are compared to those of known medicinal plants, there is comparable variation with the reported total phenolic content in the leaves of 19 mulberry varieties, with values ranging from 8.76–20.26 mg RE/g dry weight [29].

The total phenolic content is greatly influenced by environmental, climatic environments, and genetic factors [30,31,32]. In this research, the genotypes were grown under similar conditions and the samples were collected at the same time, so any differences in the total phenolic content are likely to be genotype dependent. This result showed that genotype plays an important role in the total phenolic content of kenaf leaves.

### 2.3. Total Flavonoid Content of Kenaf Leaf Extracts

Total flavonoid content for the 33 kenaf genotypes are presented in Table 1. The values ranged from 1.55–9.24 mg RE/g DW for the genotypes. Extracts from CS19 genotype showed the highest total flavonoid content (9.24 mg RE/g DW) followed by CS17 (8.81 mg RE/g DW), CS33 (7.82 mg RE/g DW), CS3 extract (7.08 mg RE/g DW), CS20 extract (7.06 mg RE/g DW), and CS28 extract (6.72 mg RE/g DW). CS22 genotype extract had the lowest total flavonoid content (1.55 mg RE/g DW).

The results of total flavonoid content of the kenaf genotypes were similar to that of a well-known beverage such as tea (*Camellia sinensis*) (3.45–5.40 RE/g DW) [33]. Although previous study found that genotype had no significant impact on the total flavonoid content [26], statistical differences in the total flavonoid content were detected among genotypes in this study. Variations in total flavonoid contents among these kenaf genotypes enable us to ease selection of genotypes that might be used as medicinal applications.

### 2.4. Antioxidant Capacities of Kenaf Leaf Extracts

The antioxidant activities of different kenaf genotypes were assessed by DPPH, ABTS, and FRAP assays, and the results are presented in Table 2.

DPPH radical scavenging activities of the extracts ranged from 20.55–79.77%, which reflects about 4-fold variation. CS2 and CS4 extracts exhibited the highest antioxidant activity with each having 79.77% of DPPH inhibition, followed by CS27 extract (79.31%), CS33 extract (78.27%), CS30 extract (75.83%), and CS9 extract (75.0%). CS18 extract exhibited the lowest antioxidant activity (20.55%). Previous study reported that the DPPH radical scavenging activity of kenaf leaves’ extracts exhibited about 65.35% of inhibition [34]. Among the 33 kenaf genotypes analyzed, 22 exhibited higher DPPH scavenging activity than the scavenging capacity of *H. sabdariffa* leaf extract reported in a previous study with a value of 65.19% [35]. Although DPPH radical scavenging activity of plant extracts depend on the solvent [36] and cultivar [37], phenolic compounds were generally considered as the source of scavenging activities of the extracts because of their hydroxyl group [38,39]. The variation in the radical scavenging activities obtained in this study can only be attributed to genotypes as the same solvent and procedure were followed in the extraction process.

The ABTS assay values varied from 56.28–88.30%, representing a lower difference than the value of DPPH assay about 2-fold. CS4 extract had the highest antioxidant activity (88.30% ABTS inhibition) followed by the CS2 extract (86.67%), CS27 extract (85.39%), CS33 (85.09), and CS16 extract (84.17%). As noted in the DPPH assay, the CS18 extract exhibited the lowest antioxidant activity (56.28%). The DPPH and ABTS assays exhibited similar results for the antioxidant activities determined in extracts of 33 kenaf genotypes. In this analysis, the values of ABTS radical inhibitions are higher than the value stated by previous study for unspecified kenaf variety [40], which had inhibition value of 25.96%. The antioxidant activity of kenaf leaf extract by the ABTS radical scavenging activity assay was also studied by other study [22] and reported an inhibition of 65.55%. The total phenolic contents may be correlated with these variations.

In FRAP assay, the pattern for reducing capacities of the 33 genotype extracts examined did not differ appreciably from their scavenging activities for DPPH and ABTS. Comparable to the results found for DPPH and ABTS assays, CS4, CS2, CS27, and CS33 extracts exhibited strong ferric-reducing capacities (5.08, 4.89, 4.80, and 4.74 mmol Fe^2+^/g DW respectively). CS18 extract had the lowest ferric reducing capacity (1.26 mmol Fe^2+^/g DW) in this study. In comparison to tea, which is a well-known source of antioxidants, kenaf extracts had higher ferric reducing capacity than white, green, and black teas [41]. In this study, the values of ferric reducing capacity of 27 genotypes are higher than the values stated by a previous study for eggplant (Purple) and marrow squash [42]. Previous studies have shown that phenolic compounds play a significant role in the reduction capacity of plant extracts [43,44]. Thus, kenaf genotypes with high levels of phenolic contents might show greater ferric reducing capacity.

### 2.5. Antibacterial Activities of Kenaf Leaf Extracts

The antibacterial activity of each extract was tested against Gram- positive (*Staphylococcus aureus*) and Gram negative (*Escherichia coli*) bacteria. As shown in Table 3, the extracts of the 33 kenaf genotypes showed antibacterial activity against two bacteria, the zones of inhibition ranging from 9.40–13.55 mm (*Staphylococcus aureus*) and from 6.15–12.67 mm (*Escherichia coli*). The Gram-negative bacteria exhibited a wider range of levels of antibacterial activity. CS5 extract exhibited the highest bacterial inhibition zone against *Staphylococcus aureus* (13.55 mm), followed by CS3 extracts (12.60 mm). CS2 extract exhib-ited the maximum zone of inhibition against *Escherichia coli* (12.67 mm), followed by CS3 extract (11.85 mm).

These findings suggest that the antibacterial effects of kenaf leaf extracts vary depending on genotype. Moreover, phenolic, flavonoid, and polysaccharide composition of the extracts are the primary inhibitors of bacteria by the alteration of membrane integrity and permeability [6,34,45,46]. The antibacterial properties of kenaf leaf extract as obtained in this study exhibited great potential as an antibacterial agent for the preservation of food.

### 2.6. Correlation between Antioxidant Capacities with Polysaccharide, Total Phenolic, and Flavonoid Content

The Pearson’s correlation analysis between antioxidant capacities with polysaccharide content, total phenolic content, and total flavonoid content is shown in Table 4. DPPH antioxidant capacities had a significant positive correlation with polysaccharide content (r = 0.893, *p* < 0.01) and total phenolic content (r = 0.850, *p* < 0.01). Strong correlation was found between ABTS antioxidant capacities with polysaccharide (r = 0.819, *p* < 0.01) and total phenolic content (r = 0.959, *p* < 0.01). Similarly, FRAP reducing capacities had a significance positive correlation with polysaccharide content (r = 0.864, *p* < 0.01) and total phenolic content (r = 0.953, *p* < 0.01). In contrast, DPPH, ABTS, and FRAP antioxidant capacities had no correlation with total flavonoid content.

According to literature, polysaccharides can significantly improve antioxidant activity and prolonged exercise performance [47]. The strong correlation between polysaccharide content and antioxidant capacity has been previously reported for other antioxidants [24,48]. In recent studies, the antioxidant capacities of kenaf leaf tea determined by DPPH and ABTS assays exhibited strong correlations with phenolic content [22,40]. Likewise, the strong correlation between FRAP assay and total phenolic of other antioxidants has been reported [44]. The number and arrangement of hydroxyl groups, as well as the existence of electron donating and electron withdrawing substituents in the ring structure of phenolics, decide their antioxidant capacity [49,50]. These results showed that polysaccharides and total phenolic content play an important role in the measured antioxidant properties of kenaf leaves, although the degree of correlation suggests that other factors could also contribute. The genotypes with the highest polysaccharide and total phenolic content also had the highest antioxidant activities determined by DPPH, ABTS, and FRAP assays.

### 2.7. Hierarchical Cluster Analysis

**Figure 1:**
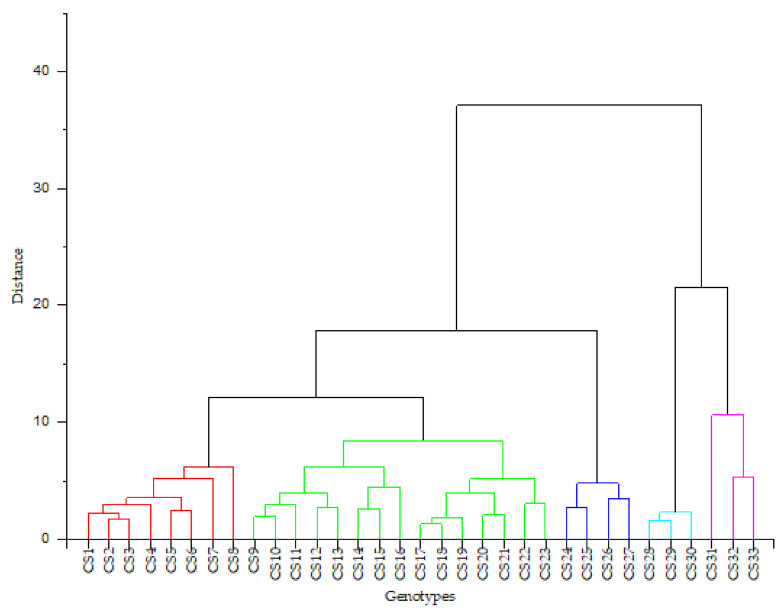
Dendrogram of hierarchical cluster analysis of the 33 kenaf genotypes according to their polysaccharide content, total phenolic content, total flavonoid content, antioxidant, and antibacterial activities using the Ward and Euclidean distance.

The hierarchical cluster analysis clustered the 33 genotypes on the basis of their bioactive compounds and antioxidant capacities’ similarities. The samples were clustered into five major groups using Ward’s method without taking into consideration the detail about the class of genotype. The results are presented in Figure 1. Cluster I contained 24.24% of the total kenaf genotypes analyzed and was characterized by high content of flavonoids. Cluster II included 45.45% of the genotypes with intermediate antioxidant activities (DPPH, ABTS, and FRAP). Cluster III accounted for 12.12% of the genotypes studied. This cluster was characterized by the highest content of total phenolic, polysaccharide, with greater antioxidant and antibacterial activities. Cluster IV accounted for 9.09% of the genotypes with intermediate content of flavonoid and low content of FRAP reducing capacity. Cluster V also accounted for 9.09% of the genotypes with the lowest content of flavonoid, phenolic, and antioxidant capacities. The analysis indicated that genotypes from cluster III could serve as potential sources of natural antioxidants.

## 3. Materials and Methods

### 3.1. Chemicals and Reagents

The 2,2′-azinobis (3-ethylbenzothiazoline-6-sulfonic acid (ABTS), 2,2-Diphenyl-1-picrylhydrazyl (DPPH), gallic acid, and rutin were obtained from Solar Bio-Science and Technology Co., Ltd. (Beijing, China). In addition, 2,4,6-tripyridyl-s-triazine (TPTZ), the regent of Folin–Ciocalteau, was purchased from Coolaber Science and Technology (Beijing, China). The highest analytical grade was used for all reagents in the analysis.

### 3.2. Plant Materials

Leaf samples from 33 kenaf genotypes were collected from Innovation experimental base of Institute of Bast Fiber Crops, Chinese Academy of Agricultural Sciences, Changsha, China, on 8 September 2020. The names of the 33 kenaf genotypes are shown in Table 5. The leaves were air dried and grinded using a small crusher (HX-200 A, Yongkang City, Xian Hardware and Pharmacy Co., Ltd., Xi’an China) and sieved using a 60- mesh sieve, labeled, and stored at room temperature for further use.

### 3.3. Preparation of Leaf Extracts

Five grams of each sample were extracted with 90% ethanol (10% *w*/*v*) by the reflux method for 2 h at 85 °C. The extracted samples were centrifuged at 4000× *g* for 15 min. The supernatants were moved to 50 mL volumetric flasks, and the procedure was repeated. Extract samples stored at −20 °C for analysis.

### 3.4. Determination of Polysaccrides

The polysaccharide content was measured following the phenol-sulfuric acid colorimetric method [51]. The sample solution was diluted 10 times with distilled water and 2 mL from which it was mixed with 1 mL of 5% phenol (*w*/*v*) and 5 mL of concentrated sulfuric acid, shaken and subsequently incubated in a boiling water bath for 15 min. The mixture was allowed to cool down to room temperature; the absorbance was then recorded at 490 nm. The standard curve was prepared with glucose (10–80 mg/L), and the results expressed as mg glucose per grams dry weight of the sample (mg glucose/g DW).

### 3.5. Determination of Total Phenolic Content

The total phenolic content was determined following the Folin–Ciocalteau colorimetric method [52]. Furthermore, 1.5 mL of Folin–Ciocalteau (20% *v*/*v*) reagent was added to 0.2 mL of each sample extract separately, mixed properly and kept for 5 min. Four milliliters of Na_2_CO_3_ (7%) were then added, and final volume made up to 10 mL with distilled water followed by incubation for 90 min in dark at room temperature. Absorbance was measured by microplate spectrophotometer at 760 nm. All determinations were calculated in triplicate. The standard curve of gallic acid (20–800 mg/L) was prepared and the results expressed as milligrams of gallic acid equivalents per grams of dry weight of the sample (mg GAE/g DW).

### 3.6. Determination of Total Flavonoid Content

The total flavonoid content of each extract was determined according to the aluminum chloride method [13]. In addition, 0.2 mL ethanolic sample extract was mixed with 4 mL of distilled water in a flask and then 0.3 mL NaNO_2_ (5%) were added to the flask and reacted for 5 min. Then, 0.3 mL AlCl_3_ (10%) was added and kept for 6 min for reaction. Then, 2 mL NaOH (4%) was added and filled with up to 10 mL with distilled water. The absorbance was measured at 510 nm. A rutin standard curve (50–400 mg/L) was prepared, and the results were reported as milligrams of rutin equivalents per grams of dry weight of the sample (mg RE/g DW). The analysis was executed in triplicate.

### 3.7. Antioxidant Capacity Determinations

#### 3.7.1. DPPH Free Radical-Scavenging Potential

The free radical-scavenging potential of the extract was determined using the Brand–Williams methods with minor modification [53]. Three milliliters of freshly prepared methanol DPPH solution (6 × 10^−5^ M) were added to 100 μL of each sample separately and the mixtures incubated in dark for 15 min at room temperature, and then the sample absorption (A sample) was recorded at 515 nm. A blank sample absorbance (A blank) containing 100 μL of methanol was also measured. Vitamin C was used as positive controls and methanol as negative control. The triplicate experiment was executed and their scavenging capacity was determined using the following equation:

inhibition (%) = [(A blank − A sample)/A blank] × 100(1)

where A blank = absorbance of the blank, and A sample = absorbance of the sample extract.

#### 3.7.2. ABTS Radical Scavenging Activity

The ABTS radical scavenging potential was determined on the basis of measuring the degree of absorbance reduction of the radical ABTS+• ion by the tested sample extracts [43]. In addition, 10 μL of sample extract and 20 μL solution of peroxidase were mixed with 170 μL solution of ABTS and allowed to react for 6 min in the dark, after which the absorption was measured using a 405 nm UV-visible spectrophotometer (A sample). By mixing 10 μL of distilled water with 20 μL solution of peroxidase and 170 μL solution of ABTS, a blank sample was prepared, and the absorbance of the blank sample was measured (A blank). Vitamin C was used as positive controls and methanol as negative. Determinations were performed in triplicate. The ABTS+• inhibition percentage was determined using the above Equation (1).

#### 3.7.3. Ferric Reducing Antioxidant Potential (FRAP)

Ferric reduction capability of extracts was determined using a modified version of the FRAP assay [54]. By adding 20 mL of acetate buffer (300 mM, pH 3.6,) and 2 mL of TPTZ (10 mM) in 40 mM hydrochloric acid and 2 mL of ferric chloride (20 mM), the working solution of the FRAP reagent was prepared. Then, the mixture was allowed to incubate at 37 °C for 30 min in a water bath. In addition, 180 μL of FRAP reagent were added to 5 μL of sample extract, and the sample absorption was estimated at 593 nm. Absorbance of a blank without a sample extract was also measured, and analyses were executed in triplicate. In order to measure the FRAP value, the difference was calculated between the sample absorbance and the blank absorbance. On the day of preparation, all solutions were used. A standard curve of ferrous sulphate (0.15–1.5 mmol/L) was used, and the results were reported in mmol ferrous ion equivalents per gram dry weight of the sample (Fe^2+^/g DW).

### 3.8. Antibacterial Activities

Antibacterial activity was determined using an agar diffusion method [55]. *Staphylococcus aureus*—A Gram-positive (ATCC 6538) and *Escherichia coli*—Gram-negative (ATCC 8739) microorganisms were used. The strains were cultivated on a Nutrient Broth medium for 24 h at 30 °C. In addition, 15 mL of nutrient agar medium were dispensed into petri dishes in a laminar airflow and allowed to solidify; then, 100 μL of bacterial suspension (10^7^ CFU/mL) were spread on the surface of the agar plates, and sterile filter paper disk (6 mm diameter) was placed on the agar plate. Furthermore, 50 mg/mL of each extract were loaded on the discs and incubated for 24 h at 37 °C. The diameter of the inhibition zones was measured and expressed in millimeters. Ethanol (80%) was used as a control [6].

### 3.9. Statistical Analysis

The values of three measurements were reported as mean ± standard deviation. Statistical analysis was conducted using SAS^®^ 9.4 software (SAS Inc., Cary, NC, USA). Duncan’s multiple comparison test was used for means separation and differences considered significant at *p* < 0.05. Hierarchical cluster analysis was performed using OriginPro^®^ version 9 software (Northampton, MA, USA). Correlation analysis was determined using SigmaPlot^®^ version 12.5 software (San Jose, CA, USA).

## 4. Conclusions

In this study, kenaf genotypes with higher phenolic and polysaccharide contents could be a significant source of natural antioxidants. The antibacterial test revealed that extracts of kenaf leaves exhibited antibacterial effects against both Gram-positive and Gram-negative bacteria. Therefore, the kenaf extracts might have the potential to serve as natural antioxidants and bio-preservatives in food industry. The results could be useful for the selection of kenaf genotypes for future genetic engineering purposes to enhance a particular bioactive compound.

## Figures and Tables

**Table 1:** Polysaccharide, total phenolic, and flavonoid content of leaf extracts from 33 kenaf genotypes (mean value ± standard deviation of three replicates).

Genotypes	Polysaccharide Content (mg Glucose/g DW)	Total Phenolic Content (mg GAE/g DW)	Flavonoid Content (mg RE/g DW)
CS1	12.12 ± 0.41k	10.91 ± 0.46ki	4.03 ± 0.49ki
CS2	15.08 ± 0.05b	19.40 ± 0.73b	5.99 ± 0.34ef
CS3	14.58 ±0.03c	14.24 ± 1.84de	7.08 ± 0.62c
CS4	16.12 ± 0.07a	21.15 ± 0.98a	4.74 ± 0.45 h–j
CS5	12.63 ± 0.01j	11.70 ± 1.02jk	6.24 ± 0.68 de
CS6	10.74 ± 0.01o	10.72 ± 0.21ki	3.32 ± 0.27mn
CS7	11.03 ± 0.06n	9.05 ± 0.10m	5.92 ± 0.13e–g
CS8	12.91± 0.03i	13.46 ± 0.15e–h	5.96 ± 0.21e–g
CS9	14.12 ± 0.06de	13.99 ± 0.15de	2.87 ± 0.13no
CS10	11.58 ± 0.06l	13.52 ± 0.42e–g	2.00 ± 0.20pq
CS11	12.48 ± 0.11j	12.83 ± 0.27f–i	2.35 ± 0.13op
CS12	11.26 ± 0.04m	12.80 ± 0.60f–i	3.32 ± 0.19mn
CS13	10.88 ± 0.05no	8.55 ± 0.75nm	3.47 ± 0.13l–n
CS14	11.05 ± 0.01n	7.70 ± 0.67no	4.37 ± 0.23jk
CS15	10.78 ± 0.02o	7.41 ± 0.41op	4.65 ± 0.37i–k
CS16	12.65 ± 0.03j	16.34 ± 0.60c	5.88 ± 0.64e–g
CS17	13.55 ± 0.01g	11.00 ± 0.33ki	8.81 ± 0.56a
CS18	6.45 ± 0.02r	6.03 ± 0.10q	5.96 ± 0.32e–g
CS19	13.39 ± 0.05gh	12.40 ± 0.56ij	9.24 ± 0.59a
CS20	12.60 ± 0.06j	12.60 ± 0.30g–j	7.06 ± 0.19c
CS21	9.79 ± 0.09p	6.64 ± 0.44pq	3.25 ± 0.10mn
CS22	5.16 ± 0.02s	6.45 ± 0.46pq	1.55 ± 0.10q
CS23	13.87 ± 0.02f	10.12 ± 0.17i	4.37 ± 0.06jk
CS24	13.34 ± 0.02h	12.52 ± 1.02h–j	3.27 ± 0.11mn
CS25	13.02 ± 0.14i	12.06 ± 0.51ij	3.55 ± 0.13lm
CS26	14.00 ± 0.05ef	12.49 ± 0.33h–j	5.56 ± 0.04fg
CS27	14.57 ± 0.20c	16.81 ± 1.47c	4.52 ± 0.26jk
CS28	12.17 ± 0.01k	10.12 ± 0.79i	6.72 ± 0.46cd
CS29	9.19 ± 0.20q	8.02 ± 0.23no	4.03 ± 1.78kl
CS30	14.20 ± 0.02d	14.54 ± 0.40d	5.30 ± 0.38ghi
CS31	12.52 ± 0.01j	13.87 ± 0.24de	5.36 ± 050fgh
CS32	13.02±0.14i	13.59 ± 0.63d–f	4.09 ± 0.48j–l
CS33	14.57±0.20c	16.63 ± 0.55c	7.82 ± 0.15b

GAE: Gallic acid equivalent; RE: rutin equivalent. Means followed by a different letter within a column indicates a significant difference (*p* < 0.05) in Duncan’s multiple range test.

**Table 2:** Antioxidant activities of leaf extracts from 33 kenaf genotypes (mean value ± standard deviation of three replicates).

Genotypes	DPPH Inhibition (%)	ABTS Inhibition (%)	FRAP (mmol Fe^2+^/g)
CS1	65.81 ± 0.64hi	69.61 ± 0.69i	3.41 ± 0.06s
CS2	79.77 ± 1.93a	86.67 ± 0.56b	4.89 ± 0.08b
CS3	71.78 ± 0.74e	80.77 ± 0.96ef	4.43 ± 0.10f
CS4	79.77 ± 1.93a	88.30 ± 0.88a	5.08 ± 0.13a
CS5	66.35 ± 1.48h	69.29 ± 1.68ij	3.48 ± 0.02r
CS6	64.22 ± 0.88jk	68.72 ± 0.60i–l	3.22 ± 0.07t
CS7	60.38 ± 1.15l	67.98 ± 0.88k–n	2.97 ± 0.07v
CS8	69.76 ± 1.30fg	68.21 ± 1.00j–m	3.98 ± 0.02k
CS9	75.01 ± 0.65c	67.95 ± 1.73k–n	4.40 ± 0.08g
CS10	69.76 ± 0.89fg	67.77 ± 1.00k–n	4.01 ± 0.03j
CS11	66.35 ± 2.03h	76.52 ± 1.72g	3.92 ± 0.10l
CS12	65.50 ± 0.88h–j	76.34 ± 0.55g	3.88 ± 0.02m
CS13	63.37 ± 0.96k	67.38 ± 1.04m–o	2.94 ± 0.02v
CS14	46.22 ± 0.80mn	66.99 ± 0.74no	2.29 ± 0.02x
CS15	45.05 ± 1.19n	65.63 ± 0.75p	2.22 ± 0.02y
CS16	73.27 ± 0.96d	84.17 ± 1.00d	4.74 ± 0.14d
CS17	68.48 ± 0.67g	68.96 ± 0.78i–k	3.42 ± 0.02s
CS18	20.55 ± 1.33p	56.28 ± 0.51s	1.26 ± 0.01z
CS19	65.39 ± 0.92h–j	74.46 ± 0.76h	3.58 ± 0.10p
CS20	66.77 ± 0.32h	76.04 ± 1.34g	3.87 ± 0.03mn
CS21	28.86 ± 0.49o	63.81 ± 0.29q	2.23 ± 0.02y
CS22	28.75 ± 0.64o	58.78 ± 0.61r	2.21 ± 0.02y
CS23	64.54 ± 0.55i–k	67.68 ± 0.60l–n	2.97 ± 0.02v
CS24	65.81 ± 0.68hi	74.82 ± 0.60h	3.85 ± 0.11n
CS25	68.90 ±1.18g	74.43 ± 0.56h	3.59 ± 0.02q
CS26	69.12 ± 1.29g	74.70 ±0.68h	3.69 ± 0.06o
CS27	79.31 ± 0.76ab	85.39 ± 1.18c	4.80 ± 0.08c
CS28	64.86 ± 0.68ij	68.42 ± 0.83i–m	3.17 ± 0.04u
CS29	47.60 ±0.55m	66.31 ± 1.78op	2.47 ± 0.02w
CS30	75.83 ± 0.74c	81.73 ± 1.27e	4.67 ± 0.07e
CS31	70.93 ± 1.56ef	80.60 ± 0.67ef	4.29 ± 0.06h
CS32	68.58 ± 1.79g	80.18 ± 1.32g	4.13 ± 0.04i
CS33	78.27 ± 1.69b	85.09 ± 0.51cd	4.74 ± 0.07d

DPPH: 2,2-Diphenyl-1-picrylhydrazyl; ABTS: 2,2′-azino-bis (3-ethylbenzothiazoline-6-sulfonic acid; FRAP: Ferric Reducing Antioxidant Potential. Means followed by the different letters within a column indicates significant difference (*p* < 0.05) in Duncan’s multiple range test.

**Table 3:** Antibacterial activity of leaf extracts from 33 kenaf genotypes against Gram positive (*Staphylococcus aureus*) and Gram-negative bacteria (*Escherichia coli*). The values are presented as mean inhibition zone (mm) ± standard deviation of three replicates including diameter of disk (6 mm).

Genotype	Zone of Inhibition (mm)	
	Bacterial Strains	
	*Staphylococcus aureus*	*Escherichia coli*
CS1	11.42 ± 0.04d	10.06 ± 0.05jk
CS2	12.05 ± 0.06c	12.67 ± 0.06a
CS3	12.60 ± 0.14b	11.85 ± 0.13b
CS4	12.43 ± 0.13b	11.18 ± 0.11c–e
CS5	13.55 ± 0.12a	6.85 ± 0.07o
CS6	12.42 ± 0.17b	10.20 ± 0.12i–k
CS7	10.54 ± 0.10gh	11.04 ± 0.61d–g
CS8	9.05 ± 0.04lm	11.47 ± 0.54b–d
CS9	11.01 ± 0.49ef	11.32 ± 0.21b–e
CS10	10.04 ± 0.19i	7.15 ± 0.28o
CS11	9.28 ± 0.42l	6.63 ± 0.05op
CS12	11.09 ± 0.10de	9.19 ± 0.20mn
CS13	12.05 ± 0.33c	8.89 ± 0.05mn
CS14	10.54 ± 0.10gh	8.74 ± 0.94n
CS15	9.62 ± 0.22jk	9.77 ± 0.26ki
CS16	10.68 ± 0.30fg	6.84 ± 0.64o
CS17	10.21 ± 0.13hi	11.22 ± 0.02c–e
CS18	9.40 ± 0.16j–l	6.15 ± 0.20p
CS19	10.54 ± 0.04gh	10.54 ± 0.14f–j
CS20	10.60 ± 0.36g	8.74 ± 0.16mn
CS21	8.60 ± 0.24n	11.22 ± 0.27c–e
CS22	7.28 ± 0.05o	6.61 ± 0.18op
CS23	8.78 ± 0.27mn	10.76 ± 0.30e–h
CS24	8.73 ± 0.11mn	10.49 ± 0.15g–j
CS25	9.31 ± 0.08kl	11.15 ± 0.07c–f
CS26	10.39 ± 0.08g–i	11.12 ± 0.15d–f
CS27	11.27 ± 0.17de	10.39 ± 0.15h–j
CS28	11.3 ± 0.24de	11.77 ± 0.37bc
CS29	11.09 ± 0.32de	10.99 ± 0.78d–g
CS30	9.70 ± 0.23j	10.30 ± 0.06i–k
CS31	9.34 ± 0.14j–l	10.56 ± 0.22f–j
CS32	11.36 ± 0.18de	9.36 ± 0.52lm
CS33	10.66 ± 0.27fg	11.59 ± 0.04b–d

Means followed by the different letter within a column indicate significant difference (*p* < 0.05) in Duncan’s multiple range test.

**Table 4:** Pearson’s correlation coefficients between polysaccharide, total phenolic content, flavonoid content, and antioxidant capacities.

Antioxidant Activities	Polysaccharide Content	Total Phenolic Content	Total Flavonoid Content
DPPH	0.893 **	0.850 **	0.253
ABTS	0.819 **	0.959 **	0.164
FRAP	0.864 **	0.953 **	0.215

** Correlation significant at *p* < 0.01.

**Table 5:** Name of the 33 kenaf genotypes used in this study.

Genotypes Code	Cultivar/Variety Name
CS1	19Sanya357
CS2	Fu Hong992
CS3	18Sanya538
CS4	18Sanya536
CS5	19Sanya599
CS6	19Sanya355
CS7	19Sanya598
CS8	WJZ1903-1
CS9	WJZ1903-2
CS10	WJZ1903-3
CS11	WJZ1903-4
CS12	WJZ-1
CS13	19WJZ-2
CS14	19WJZ-3
CS15	19WJZ-4
CS16	WMS1803
CS17	WMS1903
CS18	19BiJiA
CS19	19BiJiB
CS20	19MLM-1
CS21	19MLM-2
CS22	19MLM-3
CS23	19MLM-4
CS24	China Kenaf18
CS25	H1701
CS26	Sanya362
CS27	Sanya355
CS28	Sanya354
CS29	Sanya360
CS30	Sanya348
CS31	Sanya361
CS32	19Xin-2
CS33	19XJ-1-2

## Data Availability

Not applicable.

## References

[1] Pizzino G., Irrera N., Cucinotta M., Pallio G., Mannino F., Arcoraci V., Squadrito F., Altavilla D., Bitto A. (2017). Oxidative Stress: Harms and Benefits for Human Health. Oxid. Med. Cell. Longev..

[2] Tuttolomondo A., Simonetta I., Daidone M., Mogavero A., Ortello A., Pinto A. (2019). Metabolic and Vascular Effect of the Mediterranean Diet. IJMS.

[3] Musolino V., Gliozzi M., Nucera S., Carresi C., Maiuolo J., Mollace R., Paone S., Bosco F., Scarano F., Scicchitano M. (2019). The Effect of Bergamot Polyphenolic Fraction on Lipid Transfer Protein System and Vascular Oxidative Stress in a Rat Model of Hyperlipemia. Lipids Health Dis..

[4] Neffati N., Aloui Z., Karoui H., Guizani I., Boussaid M., Zaouali Y. (2017). Phytochemical Composition and Antioxidant Activity of Medicinal Plants Collected from the Tunisian Flora. Nat. Prod. Res..

[5] Sadeq O., Mechchate H., Es-safi I., Bouhrim M., Jawhari F., Ouassou H., Kharchoufa L., AlZain M., Alzamel N., Mohamed O. (2021). Phytochemical Screening, Antioxidant and Antibacterial Activities of Pollen Extracts from Micromeria Fruticosa, Achillea Fragrantissima, and Phoenix Dactylifera. Plants.

[6] Česonienė L., Labokas J., Jasutienė I., Šarkinas A., Kaškonienė V., Kaškonas P., Kazernavičiūtė R., Pažereckaitė A., Daubaras R. (2021). Bioactive Compounds, Antioxidant, and Antibacterial Properties of Lonicera Caerulea Berries: Evaluation of 11 Cultivars. Plants.

[7] Reddy Y.M., Kumar S.P.J., Saritha K.V., Gopal P., Reddy T.M., Simal-Gandara J. (2021). Phytochemical Profiling of Methanolic Fruit Extract of Gardenia Latifolia Ait. by LC-MS/MS Analysis and Evaluation of Its Antioxidant and Antimicrobial Activity. Plants.

[8] Nimse S.B., Pal D. (2015). Free Radicals, Natural Antioxidants, and Their Reaction Mechanisms. RSC Adv..

[9] Ruijun W., Shi W., Yijun X., Mengwuliji T., Lijuan Z., Yumin W. (2015). Antitumor Effects and Immune Regulation Activities of a Purified Polysaccharide Extracted from Juglan Regia. Int. J. Biol. Macromol..

[10] Ren D., Zhao Y., Nie Y., Lu X., Sun Y., Yang X. (2014). Chemical Composition of Pleurotus Eryngii Polysaccharides and Their Inhibitory Effects on High-Fructose Diet-Induced Insulin Resistance and Oxidative Stress in Mice. Food Funct..

[11] Wang Z., Xue R., Cui J., Wang J., Fan W., Zhang H., Zhan X. (2019). Antibacterial Activity of a Polysaccharide Produced from Chaetomium Globosum CGMCC 6882. Int. J. Biol. Macromol..

[12] Kandi S., Charles A.L. (2018). Measurement, Correlation, and Thermodynamic Properties for Solubilities of Bioactive Compound (−)-Epicatechin in Different Pure Solvents at 298.15 K to 338.15 K. J. Mol. Liq..

[13] Ryu J., Kwon S.-J., Ahn J.-W., Jo Y.D., Kim S.H., Jeong S.W., Lee M.K., Kim J.-B., Kang S.-Y. (2017). Phytochemicals and Antioxidant Activity in the Kenaf Plant (*Hibiscus cannabinus* L.). J. Plant Biotechnol..

[14] Sim Y.Y., Nyam K.L. (2021). *Hibiscus cannabinus* L. (Kenaf) Studies: Nutritional Composition, Phytochemistry, Pharmacology, and Potential Applications. Food Chem..

[15] Agbor G.A., Oben J.E., Nkegoum B., Takala J.P., Ngogang J.Y. (2005). Hepatoprotective Activity of *Hibiscus cannabinus* (Linn.) against Carbon Tetrachloride and Paracetamol Induced Liver Damage in Rats. Pak. J. Biol. Sci.

[16] Ayadi R., Hanana M., Mzid R., Hamrouni L., Khouja M.L., Salhi Hanachi A. (2016). *Hibiscus cannabinus* L.–«Kenaf»: A Review Paper. J. Nat. Fibers.

[17] Pascoal A., Quirantes-Piné R., Fernando A.L., Alexopoulou E., Segura-Carretero A. (2015). Phenolic Composition and Antioxidant Activity of Kenaf Leaves. Ind. Crop. Prod..

[18] Maganha E.G., da Costa Halmenschlager R., Rosa R.M., Henriques J.A.P., de Paula Ramos A.L.L., Saffi J. (2010). Pharmacological Evidences for the Extracts and Secondary Metabolites from Plants of the Genus Hibiscus. Food Chem..

[19] Nandagopalan V., Gritto M.J., Doss A. (2015). GC-MS Analysis of Bioactive Components of the Methanol Extract of Hibiscus Tiliaceus Linn. Asian J. Plant Sci. Res..

[20] Monti A., Alexopoulou E.E. (2013). Kenaf: A Multi-Purpose Crop for Several Industrial Applications: New Insights from the Biokenaf Project.

[21] Lim P.Y., Sim Y.Y., Nyam K.L. (2020). Influence of Kenaf (*Hibiscus cannabinus* L.) Leaves Powder on the Physico-Chemical, Antioxidant and Sensorial Properties of Wheat Bread. Food Meas..

[22] Kho K., Sim Y.Y., Nyam K.L. (2019). Antioxidant Activities of Tea Prepared from Kenaf (*Hibiscus cannabinus* L. KR9) Leaves at Different Maturity Stages. Food Meas..

[23] Tamaki Y., Kinjo K., Uechi S., Hongo F., Sameshima K., Yaga S. (2001). Cholesterol-Lowering Effect of Water-Soluble Polysaccharides from Kenaf (*Hibiscus cannabinus*) Seeds in Rats, 1. J. Jpn. Wood Res. Soc. Jpn..

[24] Guo Y.-J., Deng G.-F., Xu X.-R., Wu S., Li S., Xia E.-Q., Li F., Chen F., Ling W.-H., Li H.-B. (2012). Antioxidant Capacities, Phenolic Compounds and Polysaccharide Contents of 49 Edible Macro-Fungi. Food Funct..

[25] Yao S., Bai L., Lan Z., Tang M., Zhai Y., Huang H., Wei R. (2016). Hairy Root Induction and Polysaccharide Production of Medicinal Plant Callerya Speciosa Champ. Plant Cell Tissue Organ Cult..

[26] Ryu S.-W., Jin C.-W., Lee H.-S., Lee J.-Y., Sapkota K., Lee B.-G., Yu C.-Y., Lee M.-K., Kim M.-J., Cho D.-H. (2006). Changes in Total Polyphenol, Total Flavonoid Contents and Antioxidant Activities of *Hibiscus cannabin* Us L.. Korean J. Med. Crop Sci..

[27] Zhen J., Villani T.S., Guo Y., Qi Y., Chin K., Pan M.-H., Ho C.-T., Simon J.E., Wu Q. (2016). Phytochemistry, Antioxidant Capacity, Total Phenolic Content and Anti-Inflammatory Activity of Hibiscus Sabdariffa Leaves. Food Chem..

[28] Deng G.-F., Lin X., Xu X.-R., Gao L.-L., Xie J.-F., Li H.-B. (2013). Antioxidant Capacities and Total Phenolic Contents of 56 Vegetables. J. Funct. Foods.

[29] Yu Y., Li H., Zhang B., Wang J., Shi X., Huang J., Yang J., Zhang Y., Deng Z. (2018). Nutritional and Functional Components of Mulberry Leaves from Different Varieties: Evaluation of Their Potential as Food Materials. Null.

[30] Ghasemi Pirbalouti A., Siahpoosh A., Setayesh M., Craker L. (2014). Antioxidant Activity, Total Phenolic and Flavonoid Contents of Some Medicinal and Aromatic Plants Used as Herbal Teas and Condiments in Iran. J. Med. Food.

[31] Min K., Freeman C., Kang H., Choi S.-U. (2015). The Regulation by Phenolic Compounds of Soil Organic Matter Dynamics under a Changing Environment. BioMed Res. Int..

[32] Deng M., Deng Y., Dong L., Ma Y., Liu L., Huang F., Wei Z., Zhang Y., Zhang M., Zhang R. (2018). Effect of Storage Conditions on Phenolic Profiles and Antioxidant Activity of Litchi Pericarp. Molecules.

[33] Gonbad R.A., Afzan A., Karimi E., Sinniah U.R., Swamy M.K. (2015). Phytoconstituents and Antioxidant Properties among Commercial Tea (Camellia Sinensis L.) Clones of Iran. Electron. J. Biotechnol..

[34] Adnan M., Oh K.K., Azad M.O.K., Shin M.H., Wang M.-H., Cho D.H. (2020). Kenaf (*Hibiscus cannabinus* L.) Leaves and Seed as a Potential Source of the Bioactive Compounds: Effects of Various Extraction Solvents on Biological Properties. Life.

[35] Subhaswaraj P., Sowmya M., Bhavana V., Dyavaiah M., Siddhardha B. (2017). Determination of Antioxidant Activity of Hibiscus Sabdariffa and Croton Caudatus in Saccharomyces Cerevisiae Model System. J. Food Sci. Technol..

[36] Xie J., Schaich K.M. (2014). Re-Evaluation of the 2,2-Diphenyl-1-Picrylhydrazyl Free Radical (DPPH) Assay for Antioxidant Activity. J. Agric. Food Chem..

[37] Boneza M.M., Niemeyer E.D. (2018). Cultivar Affects the Phenolic Composition and Antioxidant Properties of Commercially Available Lemon Balm ( *Melissa officinalis* L.) Varieties. Ind. Crop. Prod..

[38] Polumackanycz M., Sledzinski T., Goyke E., Wesolowski M., Viapiana A. (2019). A Comparative Study on the Phenolic Composition and Biological Activities of Morus Alba L. Commercial Samples. Molecules.

[39] Duan L., Guo L., Dou L., Yu K.-Y., Liu E.-H., Li P. (2014). Comparison of Chemical Profiling and Antioxidant Activities of Fruits, Leaves, Branches, and Flowers of *Citrus Grandis* ‘Tomentosa’. J. Agric. Food Chem..

[40] Sim Y.Y., Jess Ong W.T., Nyam K.L. (2019). Effect of Various Solvents on the Pulsed Ultrasonic Assisted Extraction of Phenolic Compounds from *Hibiscus cannabinus* L. Leaves. Ind. Crop. Prod..

[41] Al-Obaidi R., Sahib D. (2015). Determination of Antioxidants Activity in Tea Extract. Am. J. Biochem..

[42] Wong C.-C., Li H.-B., Cheng K.-W., Chen F. (2006). A Systematic Survey of Antioxidant Activity of 30 Chinese Medicinal Plants Using the Ferric Reducing Antioxidant Power Assay. Food Chem..

[43] Kalam Azad M.O., Jeong D.I., Adnan M., Salitxay T., Heo J.W., Naznin M.T., Lim J.D., Cho D.H., Park B.J., Park C.H. (2019). Effect of Different Processing Methods on the Accumulation of the Phenolic Compounds and Antioxidant Profile of Broomcorn Millet (*Panicum miliaceum* L.) Flour. Foods.

[44] Huang D., Ou B., Prior R.L. (2005). The Chemistry behind Antioxidant Capacity Assays. J. Agric. Food Chem..

[45] Ben Yakoub A.R., Abdehedi O., Jridi M., Elfalleh W., Nasri M., Ferchichi A. (2018). Flavonoids, Phenols, Antioxidant, and Antimicrobial Activities in Various Extracts from Tossa Jute Leave (*Corchorus olitorus* L.). Ind. Crop. Prod..

[46] He F., Yang Y., Yang G., Yu L. (2010). Studies on Antibacterial Activity and Antibacterial Mechanism of a Novel Polysaccharide from Streptomyces Virginia H03. Food Control.

[47] Rosset R., Lecoultre V., Egli L., Cros J., Rey V., Stefanoni N., Sauvinet V., Laville M., Schneiter P., Tappy L. (2017). Endurance Training with or without Glucose-Fructose Ingestion: Effects on Lactate Metabolism Assessed in a Randomized Clinical Trial on Sedentary Men. Nutrients.

[48] Zhang A., Shen Y., Cen M., Hong X., Shao Q., Chen Y., Zheng B. (2019). Polysaccharide and Crocin Contents, and Antioxidant Activity of Saffron from Different Origins. Ind. Crop. Prod..

[49] Biswas A., Dey S., Li D., Liu Y., Zhang J., Huang S., Pan G., Deng Y. (2020). Comparison of Phytochemical Profile, Mineral Content, and *In Vitro* Antioxidant Activities of *Corchorus Capsularis* and *Corchorus Olitorius* Leaf Extracts from Different Populations. J. Food Qual..

[50] Sridhar K., Charles A.L. (2019). In Vitro Antioxidant Activity of Kyoho Grape Extracts in DPPH and ABTS Assays: Estimation Methods for EC50 Using Advanced Statistical Programs. Food Chem..

[51] DuBois M., Gilles K.A., Hamilton J.K., Rebers P.A., Smith F. (1956). Colorimetric Method for Determination of Sugars and Related Substances. Anal. Chem..

[52] Jin C.W., Ghimeray A.K., Wang L., Xu M.L., Piao J.P., Cho D.H. (2013). Far Infrared Assisted Kenaf Leaf Tea Preparation and Its Effect on Phenolic Compounds, Antioxidant and ACE Inhibitory Activity. J. Med. Plants Res..

[53] Brand-Williams W., Cuvelier M.E., Berset C. (1995). Use of a Free Radical Method to Evaluate Antioxidant Activity. LWT-Food Sci. Technol..

[54] Benzie I.F.F., Strain J.J. (1996). The Ferric Reducing Ability of Plasma (FRAP) as a Measure of “Antioxidant Power”: The FRAP Assay. Anal. Biochem..

[55] Adnan M., Azad M.O.K., Madhusudhan A., Saravanakumar K., Hu X., Wang M.-H., Ha C.D. (2020). Simple and Cleaner System of Silver Nanoparticle Synthesis Using Kenaf Seed and Revealing Its Anticancer and Antimicrobial Potential. Nanotechnology.

